# A Multisystem Presentation of West Nile Neuroinvasive Disease: Lessons in Diagnostic Complexity

**DOI:** 10.7759/cureus.115298

**Published:** 2026-08-27

**Authors:** Carrine S Kogulan, Philip Yusuf, Oluwafemi Ajibola

**Affiliations:** 1 Internal Medicine, Kansas City University, Joplin, USA; 2 Neurology, Kansas City University, Joplin, USA; 3 Pulmonary and Critical Care Medicine, Freeman Health System, Joplin, USA

**Keywords:** hypoxic respiratory failure, neuroinvasive disease, subacute thyroiditis, tick-born encephalitis, west nile encephalitis

## Abstract

West Nile virus (WNV) is an uncommon but serious case of encephalitis and meningitis, often presenting with nonspecific symptoms that can mimic a wide range of infectious, metabolic, and endocrine disorders. We present the case of a 61-year-old male with multiple comorbidities who arrived with 5 days of fever, chills, profuse diarrhea, black stools, and worsening confusion. Initial evaluation in the emergency department revealed hyponatremia, transaminitis, and concern for tick-borne illness, prompting empiric doxycycline. However, the patient denied any tick bites. Given his known thyroid nodule and Burch-Wartofsky screening score of 70, he was empirically treated for thyroid storm despite laboratory evidence of subacute hypothyroidism.

During hospitalization, our patient developed acute hypoxic respiratory failure requiring emergent intubation, as well as second-degree heart block and supraventricular tachycardia following propranolol administration. Neurologic workup revealed moderate encephalopathy on EEG and an opening pressure of 36 cm H2O on lumbar puncture. CSF examination revealed pleocytosis with mixed neutrophilic and lymphocytic predominance. Despite broad-spectrum antibiotic coverage, initial testing, which included a tick panel, Monospot, respiratory studies, and viral PCR assays, was insignificant.

On hospital day eight, CSF serologies were positive for WNV IgM with negative IgG, confirming acute WNV neuroinvasive disease presenting as meningitis. This case highlights the diagnostic complexity of WNV infection, which may initially resemble tick-borne illness, endocrine crisis, or bacterial meningitis. Early consideration of WNV in patients with unexplained encephalopathy, GI symptoms, hyponatremia, and CSF lymphocytic pleocytosis, especially during mosquito season, may reduce delays in diagnosis and prevent unnecessary interventions.

## Introduction

West Nile virus (WNV) is a mosquito-borne flavivirus with human, equine, and avian hosts [1]. Birds are the natural reservoir of the virus, with Culex sp. mosquitoes being the transmitters. In North American countries, the virus has manifested as meningitis, encephalitis, and poliomyelitis, causing high rates of morbidity and mortality [2]. WNV is a leading cause of arthropod-borne viral neuroinvasive disease in the US [3]. There are limited reports of West Nile meningoencephalitis in the United States, as the virus is primarily indigenous to Africa, Asia, Europe, and Australia [2].

In humans, the virus’s clinical presentation can range from asymptomatic (implicated in 80% of infections) to encephalitis, meningitis, and poliomyelitis-like disease, presented as acute flaccid paralysis [1,2]. Thus, the virus can be implicated in high rates of mortality and morbidity, especially with increasing age. Most symptomatic patients will have mild disease courses with prodrome, including headache, myalgias, nausea, vomiting, chills, and sometimes a papular rash on the arms, legs, or trunk. Of the remaining 20% of symptomatic patients, only 5% develop neurologic manifestations [4]. West Nile encephalitis and meningitis present with rapid onset of headache, photophobia, back pain, confusion, and continued fever. In contrast, WNV poliomyelitis-like syndrome presents as acute onset of areflexia and absent weakness characterized by loss of pain sensation [5].

The diagnosis of patients with neurological manifestations includes cerebrospinal fluid (CSF) examination to rule out stroke, myopathy, and Guillain-Barré syndrome (GBS). Also, imaging can be helpful in diagnosis, but is not very sensitive for WNV. Computed tomography (CT) scanning can reveal pre-existing lesions and changes in brain tissue but will rarely reveal signs of central nervous system (CNS) inflammation. However, magnetic resonance imaging (MRI) will show enhancement of the leptomeninges or periventricular areas, consistent with inflammation and encephalitis in 30% of West Nile neurological patients. CSF findings in West Nile meningoencephalitis can include mild pleocytosis (30-100 cells/µL; range 0-1800 cells/µL) with lymphocytic predominance; elevated protein concentration of around 80-105 mg/dL (rarely, up to 1900 mg/dL); and normal glucose concentrations. Moderate leukocytosis can present with counts up to 30,000 cells/ µL, though only occurring in less than 50% of patients [1]. In West Nile encephalitis, muscle weakness is a key clinical feature, with approximately 10% of patients progressing to flaccid paralysis. This is associated with an absence of reflexes and a high incidence of respiratory failure [6]. We present a case of West Nile meningoencephalitis in which nonspecific multisystem findings and multiple competing diagnoses delayed definitive diagnosis.

## Case presentation

**Figure 1:**
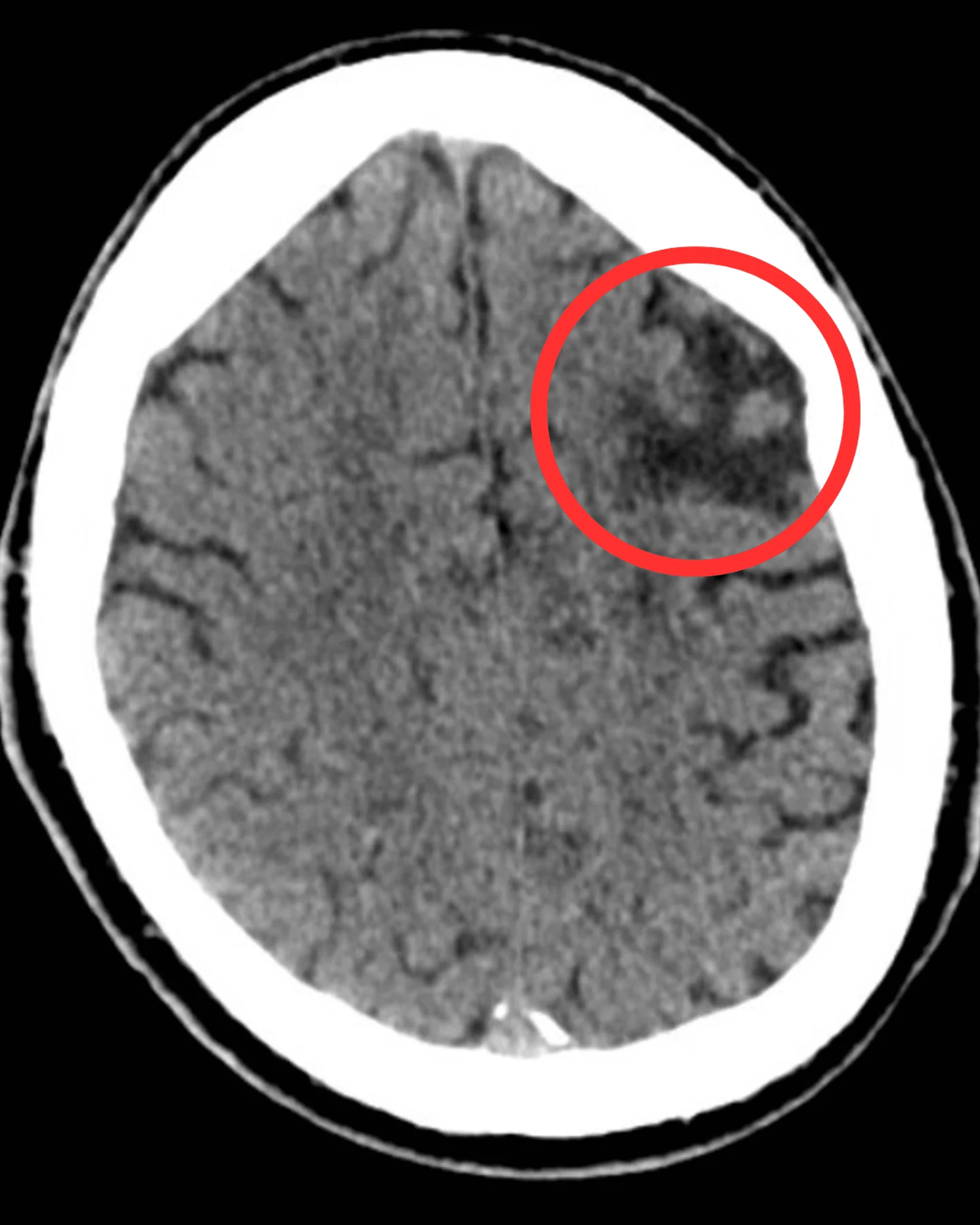
Brain CT on admission significant for stable areas of encephalomalacia in the left frontal and parietal lobes This finding delayed the diagnosis.

A 61-year-old male with a past medical history of type 2 diabetes, stroke, chronic obstructive pulmonary disease (COPD), hypertension (HTN), and hyperlipidemia presented to the emergency department with the chief complaint of black stools and fever that he had had for the last 5 days, which had progressed to profuse diarrhea, fever, and chills. The patient was notably confused upon presentation to the ED. He had also been having intermittent sharp lower abdominal pain but denied nausea and vomiting. He had presented to the ED four days before this visit, with similar symptoms. Labs revealed mild hyponatremia, and he was told to take acetaminophen as needed after he was discharged from the hospital. He denied any sick contacts, upper respiratory symptoms, or recent tick bites. On admission (Figure 4), his labs revealed hyponatremia at 126 and elevated liver function tests (LFTs) (Table 1). He was empirically started on doxycycline for presumptive tick-borne illness, and a subsequent tick panel and Monospot test were sent for evaluation. Additional labs showed potassium at 4, creatinine at 0.76, BUN at 7, elevated blood glucose at 165, plasma calcium at 8.2, and albumin at 3.1. He also had transaminitis with aspartate aminotransferase (AST) of 140 and alanine aminotransferase (ALT) of 73, but no leukocytosis. Urinalysis was not significant for a urinary tract infection (UTI). After admission, he was continued on doxycycline and started on metronidazole and azithromycin for possible infectious diarrhea. Monospot testing was negative. Head CT was obtained for altered mental status and prolonged fever, which showed no acute changes (Figure 1).

**Table 1:** Key laboratory and cerebrospinal fluid findings during hospitalization Labs indicated tickborne disease with elevated LFTs, hyponatremia, and leukopenia. WBC, white blood cells; AST, aspartate aminotransferase; ALT, alanine aminotransferase; CSF, cerebrospinal fluid; TSH, thyroid-stimulating hormone; BUN, blood urea nitrogen; LFT, liver function test

Lab	Patient’s Value	Normal Range
WBC	4.4	4.5-11 x10³/µL
Sodium (Na+)	126	135-145 mEq/L
Potassium (K+)	4	3.5-5.0 mEq/L
Calcium	8.2	8.4-10.2 mg/dL
AST	140	12-38 U/L
ALT	73	10-40 U/L
CSF glucose	128	40-70 mg/dL
CSF total proteins	197	<40 mg/dL
CSF WBC	140	0-5/mm³
TSH	0.25	0.4-4.0 μU/mL
Glucose	165	70-110 mg/dL
Creatinine	0.76	0.6-1.2 mg/dL
BUN	7	7-18 mg/dL
Albumin	3.1	3.5-5.5 g/dL

With the patient’s symptoms and history of a thyroid nodule and goiter, he was worked up for a potential thyroid storm. He had a positive score on the Burch-Wartofsky screening scale [7], scoring 70, with greater than 45 indicating a thyroid storm. He was started on methimazole and propranolol for the presumed thyroid storm. Additionally, he had orthostatic hypotension, so fluid resuscitation was continued with normal saline. Based on laboratory values, his thyroid-stimulating hormone (TSH) indicated subacute hypothyroidism, but the Burch-Wartofsky point scale was highly suggestive of a thyroid storm.

**Figure 2:**
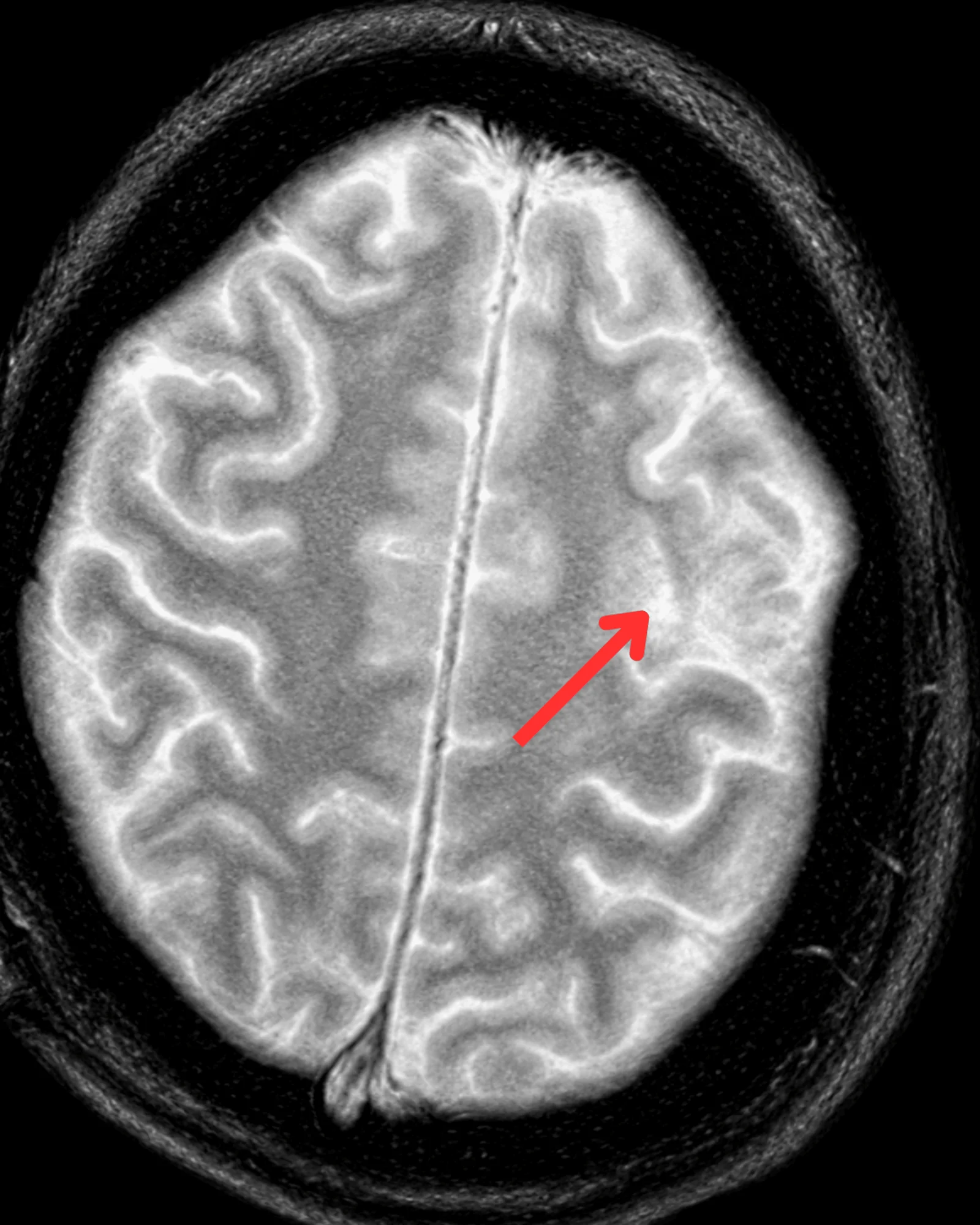
Brain MRI on admission showing no acute intracranial pathology, with findings limited to chronic changes consistent with prior infarction. Imaging may be normal in early West Nile encephalitis.

On the second day of admission, our patient had a rapid response for altered mental status and hypoxia, requiring emergency intubation. Subsequently, the patient was found to have a second-degree heart block following the propranolol dosing for the possible thyroid storm. Neurology was consulted for worsening altered mental status, for which they suggested repeating the MRI of the brain without contrast, EEG, and proceeding with a lumbar puncture for CSF analysis. Initial MRI of the brain revealed areas of encephalomalacia in the left frontal and parietal lobes and evidence of previous stroke but no acute changes (Figure 2).

Repeated labs on day 3 of admission revealed improvement of thyroid function tests, making a thyroid storm less likely. At this time, lumbar puncture was pending, as clopidogrel and apixaban needed to be held for three days, and infectious disease was consulted. EEG revealed moderate encephalopathy due to continuous generalized slowing, which was nonspecific in etiology, as blood cultures and lumbar puncture were still pending. On neurological examination, the patient was intubated and had been off sedation for approximately 10 minutes. He remained unresponsive to verbal and noxious stimuli and did not demonstrate spontaneous movement of any extremity. There was no nuchal rigidity. Pupils were equal, round, and reactive to light, although sluggish. Corneal reflexes were absent. Gag and cough reflexes were present, with grimacing elicited during testing. Given his impaired level of consciousness and intubated status, formal assessment of orientation, speech, motor strength, sensation, coordination, and gait could not be performed. All other organ systems had no significant findings. Chest X-ray revealed left lower lobe consolidations secondary to possible hospital-acquired pneumonia (HAP) with a small, associated pleural effusion.

On the fourth day of admission, bronchoscopy was performed at the bedside, and bronchoalveolar lavage (BAL) samples were sent for culture. The patient was continued on meningitis prophylaxis antibiotics, including meropenem, acyclovir, and vancomycin. Lumbar puncture was done at the bedside, with clear liquid collected. The opening pressure was 36. Infectious disease confirmed that the lumbar puncture was consistent with meningitis, potentially viral meningitis. There was leukocytosis of 59 with a differential indicating 47% neutrophils and 47% lymphocytes. Gram stain of the BAL showed some yeast and gram-positive cocci.

On the fifth day of admission, the patient developed supraventricular tachycardia, for which he was given adenosine without sustained improvement in heart rate. He was given an additional dose of IV diltiazem and started on a diltiazem drip to combat the arrhythmia. Continuous EEG showed no seizure activity. Infectious disease had recommended that he be switched to ampicillin and ceftriaxone for meningitis and HAP coverage. Our patient remained stable on the vent and was monitored by neurology, infectious disease, and cardiology.

On the eighth day of admission, the results of the lumbar puncture cultures were ready to be identified. Spinal tap testing for viruses revealed negative results for varicella-zoster virus, cytomegalovirus, and enterovirus per PCR testing. Subsequent testing for fungal antibodies showed no detection of the cryptococcus antigen, but confirmed WN with IgG values <1.30 and IgM values at 4.59. Additional testing for meningitis-related microbes was negative. Once acute WN meningeal encephalitis was confirmed to be the diagnosis, our treatment plan was more specific to our patient’s presentation.

**Figure 3:**
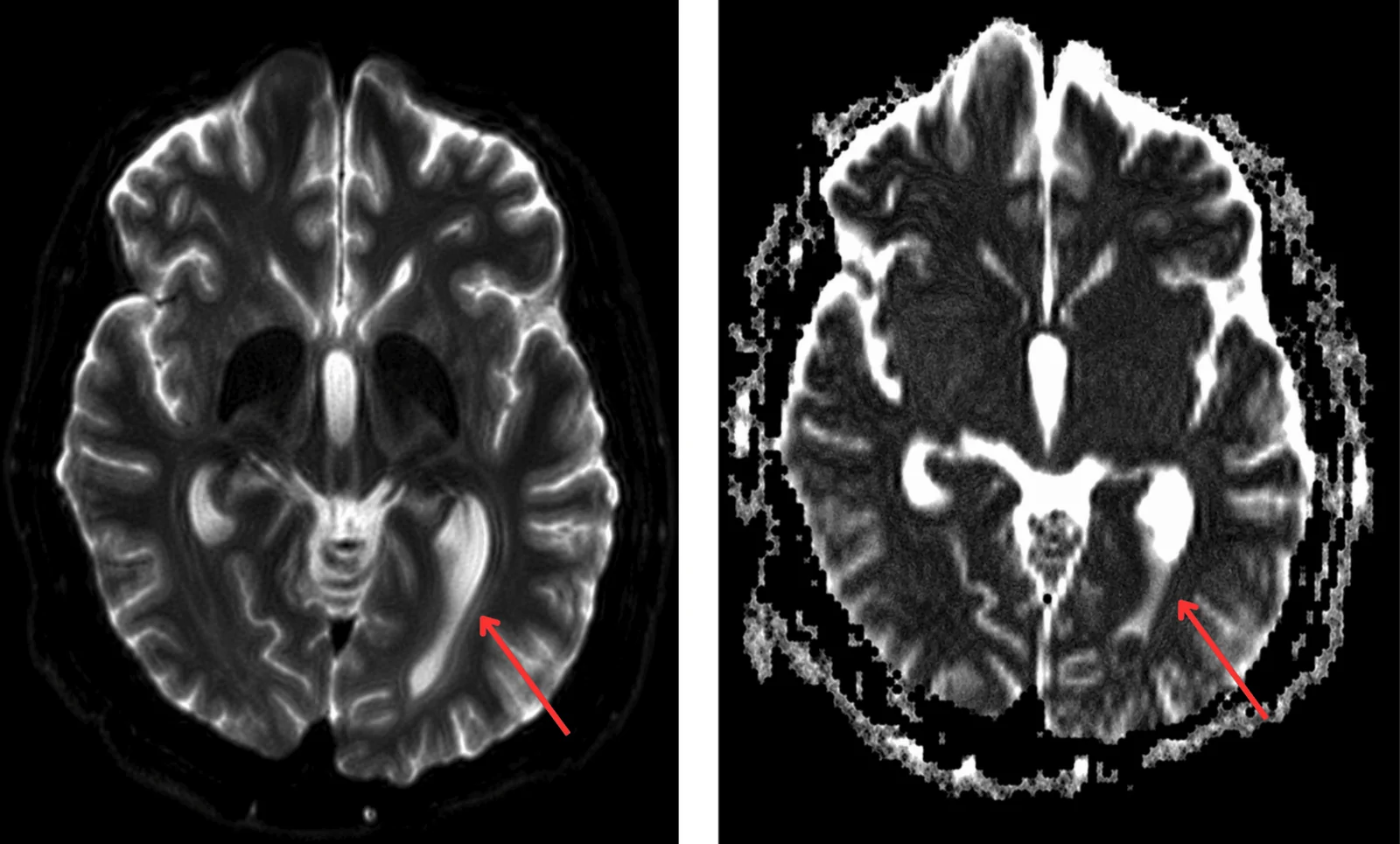
Repeat brain MRI demonstrating persistent tiny foci of restricted diffusion along the occipital horns of the lateral ventricles, slightly more prominent on the left. Diffusion-weighted imaging (DWI) is shown on the left, with the corresponding apparent diffusion coefficient (ADC) map on the right. Red arrows indicate the area of abnormal diffusion.

At the time of diagnosis, our patient was having increasing right arm weakness with flexion, for which we ruled out acute stroke with MRI and started the patient on IVIG and continued steroids. Neurological examination was limited by intubation and sedation. The patient demonstrated some eye opening and head turning. Pupils were equal, round, and reactive to light, although sluggish, and the tongue protruded at midline. He moved all extremities spontaneously, with mild right upper-extremity weakness in flexion compared with the left. Minimal motor response to noxious stimuli was observed, with slight flexion of the extremities. Formal MRC motor strength grading, deep tendon reflexes, plantar responses, sensory examination, coordination, and gait could not be reliably assessed at that time because of sedation. These components were not subsequently documented in sufficient detail to permit retrospective reporting. Repeat MRI showed a punctate lesion in the occipital horn (Figure 3) that was likely secondary to this patient’s meningoencephalitis. Continued limb weakness was concerning, as it is not a normal finding consistent with meningoencephalitis, though MRI of the cervical spine was negative for any acute spinal cord abnormality.

Over the next couple of days, our patient remained stable on the ventilator with increasing neurologic function. The patient was able to respond to short questions with nods and verbally “yes” and “no”. The family wished to proceed with a tracheostomy and percutaneous endoscopic gastrostomy (PEG) tube placement for increasing nutritional needs and long-term mechanical ventilation. IVIG was discontinued, as neurology recommended a five-day course. Infectious disease was unsure whether IVIG would have a significant effect in the treatment of West Nile meningoencephalitis. The patient continued on tube feeds for nutrition and was being monitored in the intensive care unit. He was working with the occupational and physical therapists to regain motor strength after showing improvement in neurological function. In the meantime, social services were working to find our patient a placement in a long-term care facility, as he would need continual care for his diagnoses. Three days post PEG tube placement and one day post tracheostomy, our patient was accepted and transferred to a long-term care facility.

Table 2 lists the clinical course and diagnostic progression of West Nile meningoencephalitis in our patient.

**Table 2:** Clinical course and diagnostic progression of West Nile meningoencephalitis AST, aspartate aminotransferase; ALT, alanine aminotransferase; CSF, cerebrospinal fluid; EEG, electroencephalogram; IVIG, intravenous immunoglobulin; LP, lumbar puncture; PEG, percutaneous endoscopic gastrostomy; SVT, supraventricular tachycardia; WNV, West Nile virus

Hospital Day	Phase	Clinical Status	Key Diagnostic Findings	Management	Diagnostic Significance
Day −4	Prior ED visit	Fever, melena, diarrhea	Mild hyponatremia, Na 130	Supportive care; discharged	No diagnosis
Day 0	Admission	5 days of melena/diarrhea; fever, chills, confusion, abdominal pain	Na 126; AST 140; ALT 73; CT head: no acute change	Doxycycline; metronidazole + azithromycin added	Infectious process considered
Days 1–3	Clinical deterioration	Worsening encephalopathy; hypoxia	Burch-Wartofsky 70; MRI: chronic infarcts only; EEG: moderate encephalopathy	Methimazole + propranolol; intubation; LP delayed due to anticoagulation	Thyroid storm considered; persistent unexplained encephalopathy
Day 4	CNS evaluation	Persistent encephalopathy	LP: opening pressure 36 cm H₂O; CSF pleocytosis	Empiric meropenem, vancomycin, acyclovir	Meningitis/encephalitis suspected
Day 5	ICU course	SVT	Continuous EEG: no seizures	Adenosine → diltiazem; antibiotics de-escalated	Neurologically stable on ventilator
Day 8	Definitive diagnosis	New right arm weakness	CSF WNV IgM 4.59 positive; IgG negative; MRI negative for acute stroke	IVIG + corticosteroids	Acute West Nile meningoencephalitis
Day 9+	Recovery/disposition	Gradual neurologic improvement	Able to nod and answer yes/no	Completed 5-day IVIG; PEG + tracheostomy	Transferred to long-term care

## Discussion

WNV is a leading cause of mosquito-borne neuroinvasive disease in the United States. Early infection with this virus often presents with generalized symptoms such as fever, fatigue, headaches, myalgia, and gastrointestinal complaints, which can be difficult to distinguish from other common infections and pathologies [8-10]. Most infections are asymptomatic, but fewer than 1% progress to meningitis, encephalitis, or acute flaccid paralysis, particularly among older adults or patients with chronic medical conditions, carrying a fatality rate of approximately 10% [8,9,11].

Our patient presented with hyponatremia, transaminitis, and altered mental status findings that broadened the initial differential diagnosis rather than supporting a specific diagnosis. The combination of fever, hyponatremia, and transaminitis is frequently seen in several tick-borne infections, contributing to the initial diagnostic uncertainty. Although fever and encephalopathy are common in WNV infection, atypical presentations have been reported and can delay diagnosis [8,11]. Presentations of WNV-related illness can sometimes be unrecognizable clinically [8]. In a follow-up study of viremic blood donors who later developed WNV fever, only 38% sought medical care, 2% were hospitalized, and only 5% of those seeking care were correctly diagnosed with WNV infection, once again highlighting the diagnostic challenge [12]. This challenge is especially heightened when the disease progresses to encephalopathy, as in our case.

The coexistence of a suspected thyroid storm further complicated the clinical picture, as infection is a recognized precipitating factor for thyroid storms, and severe thyrotoxicosis itself can produce encephalopathy, arrhythmias, and multiorgan dysfunction [13,14]. In this case, systemic infection likely contributed to metabolic instability and cardiovascular complications, leading to empiric treatment for thyroid storm despite laboratory findings inconsistent with hyperthyroidism.

The pathogenesis of WNV neuroinvasion involves hematogenous spread, disruption of the blood-brain barrier, and direct neuronal infection [15]. Advanced age and chronic diseases, such as diabetes and hypertension, increase the risk of severe neuroinvasive disease, consistent with our patient’s comorbidities, as summarized in epidemiologic studies of WNV [9]. Diagnosis requires clinical suspicion supported by cerebrospinal fluid testing. CSF typically demonstrates lymphocytic pleocytosis with elevated protein levels, and the detection of WNV-specific IgM in CSF confirms a neuroinvasive infection [8]. The Centers for Disease Control and Prevention (CDC) also recommends considering West Nile virus in the differential diagnosis of patients with unexplained encephalitis or meningitis [9,10].

In our patient, the increased opening pressure, CSF pleocytosis, elevated protein, negative bacterial and fungal studies, and positive WNV IgM were most consistent with viral meningitis. Although our patient had an elevated CSF glucose, this was likely related to concurrent serum hyperglycemia rather than the underlying CNS infection. Management remains supportive because no antiviral therapy has demonstrated consistent clinical benefit, and many survivors experience persistent neurologic deficits [16].

In an older patient with fever, GI symptoms, encephalopathy, hyponatremia, transaminitis, negative early infectious workup, and CSF pleocytosis during mosquito season, WNV neuroinvasive disease should remain high in the differential even when imaging is initially unrevealing.

This case illustrates how WNV infection can mimic thyroid storm, tick-borne illness, and bacterial meningitis, emphasizing the importance of maintaining a wide differential diagnosis when evaluating patients with similar presentations of encephalopathy. Early consideration of WNV may help prevent diagnostic delays and unnecessary empiric treatments when initial clinical and laboratory findings suggest alternative etiologies.

## Conclusions

Clinicians should consider WNV in older or medically complex patients presenting with unexplained fever, encephalopathy, gastrointestinal symptoms, hyponatremia, and CSF pleocytosis during mosquito season, even when early imaging and initial infectious testing are unrevealing.

WNV remains an underdiagnosed cause of neuroinvasive disease, due to its nonspecific early presentation and overlapping with other infectious and metabolic conditions, which can lead to delays in diagnosis and appropriate supportive management. While treatment remains largely supportive, early recognition may reduce unnecessary diagnostic testing, help avoid inappropriate empiric therapies, and facilitate timely supportive care.
